# Tumor Mutation Burden and Immune Invasion Characteristics in Triple Negative Breast Cancer: Genome High-Throughput Data Analysis

**DOI:** 10.3389/fimmu.2021.650491

**Published:** 2021-04-21

**Authors:** Chundi Gao, Huayao Li, Cun Liu, Xiaowei Xu, Jing Zhuang, Chao Zhou, Lijuan Liu, Fubin Feng, Changgang Sun

**Affiliations:** ^1^College of First Clinical Medicine, Shandong University of Traditional Chinese Medicine, Jinan, China; ^2^College of Basic Medical, Shandong University of Traditional Chinese Medicine, Jinan, China; ^3^Department of Oncology, Weifang Traditional Chinese Hospital, Weifang, China; ^4^Qingdao Academy of Chinese Medical Sciences, Shandong University of Traditional Chinese Medicine, Qingdao, China

**Keywords:** triple-negative breast cancer, tumor mutation burden, immune infiltration, GSEA pathway analysis, drug sensitivity

## Abstract

In recent years, the emergence of immunotherapy has provided a new perspective for the treatment and management of triple-negative breast cancer (TNBC). However, the relationship between tumor mutation burden (TMB) and immune infiltration and the prognosis of TNBC remains unclear. In this study, to explore the immunogenicity of TNBC, we divided patients with TNBC into high and low TMB groups based on the somatic mutation data of TNBC in The Cancer Genome Atlas (TCGA), and screened out genes with mutation rate ≥10. Then, Kaplan-Meier survival analysis revealed that the 5-year survival rate of the high TMB group was much higher than that of the low TMB group and the two groups also showed differences in immune cell infiltration. Further exploration found that the FAT3 gene, which displays significant difference and a higher mutation rate between the two groups, is not only significantly related to the prognosis of TNBC patients but also exhibits difference in immune cell infiltration between the wild group and the mutant group of the FAT3 gene. The results of gene set enrichment analysis and drug sensitivity analysis further support the importance of the FAT3 gene in TNBC. This study reveals the characteristics of TMB and immune cell infiltration in triple-negative breast cancer and their relationship with prognosis, to provide new biomarkers and potential treatment options for the future treatment of TNBC. The FAT3 gene, as a risk predictor gene of TNBC, is considered a potential biological target and may provide new insight for the treatment of TNBC.

## Introduction

In recent years, immune checkpoint inhibitors have been used in immunotherapy for cancer treatment and have attracted worldwide attention. Programmed death-1 (PD-1) and its ligand (PD-L1) inhibitors are used as immune checkpoint monoclonal antibodies, and the reports on the breadth, depth, and durability of their responses are few. These inhibitors have become a hot topic in the research of various tumor immunotherapies, including breast cancer (1). Tumor mutation burden (TMB), one of the biomarkers that has a greater correlation with the efficacy of the PD-1 antibody, can reflect the total number of mutations carried by tumor cells and has been proven to play an important role in the treatment of a variety of cancers with mutations, including breast cancer and rectal cancer (2–4). It is worth noting that the corresponding neoantigen levels in tumor cells with high TMB are correspondingly higher and can further enhance the immunogenicity of the tumor, help the immune system recognize the tumor, and stimulate the proliferation and anti-tumor response of anti-tumor T cells, thereby improving the patient’s response to cancer immunotherapy (5).

Triple-negative breast cancer (TNBC) is associated with a severely poor prognosis due to its aggressive phenotype and lack of biomarker-driven targeted therapy. Studies have found that compared with other subtypes of breast cancer, such as HER-2 positive breast cancer, TNBC exhibits more immunogenic characteristics, which is mainly reflected in the higher proportion of tumor infiltrating lymphocytes (6), and thus may be a useful marker for tumor immunotherapy. Many clinical studies have shown that PD-L1 is expressed in the tumor tissues of patients with TNBC (7, 8), and clinical studies focusing on immune checkpoint therapy have also found that certain clinical effects have been achieved in TNBC treatment (9). However, the role of TMB— a biomarker that has greater correlation with the efficacy of PD-1 antibodies— in the immunogenicity of TNBC remains unclear, and further studies are needed.

Studies have shown that the mutation load or the number of mutations in tumor DNA is an adequate marker for predicting the response of checkpoint inhibitor immunotherapy to different cancers (10, 11). The prognostic role of TMB and relationship between TMB and immune infiltrate varied from different types of cancers (12, 13), and high TMB is closely related to clinical benefits and prolonged survival of patients (14–16). In recent years, with the increase in biomarker-driven therapy for TNBC management, TMB, a quantitative indicator of tumor antigenicity, has received increasing attention. Whether TMB can help expand the clinical treatment options of patients diagnosed with this subtype of breast cancer to ultimately improve clinical outcomes and survival rate is the focus of the current research.

In this study, we used the TNBC-related mutation and transcriptome data from the cancer genome map (TCGA) database. Firstly, the genes with mutation rate ≥10 in TNBC samples were screened, and the samples were divided into high and low TMB groups according to the median TMB. Then the target genes were screened according to the differential expression analysis of the two groups of highly mutated genes, and the effects of target genes and high and low TMB groups on the prognosis of the patients were discussed according to the clinical characteristics of the target patients. Finally, the results of gene enrichment analysis and drug sensitivity analysis further support the importance of target genes in TNBC. The relationships that this study attempts to clarify include: TNBC-related mutation gene, high and low TMB and clinical outcome, enrichment of immune cells and pathways affected by mutation and TMB, target gene and drug sensitivity.

## Materials and Methods

### Data Acquisition

Transcriptome, mutation, and clinical trait data related to TNBC were obtained from TCGA (https://cancergenome.nih.gov/) (17). Among the data, 116 cases of triple-negative breast cancer samples with complete clinical prognosis information were obtained, while 101 samples containing mutation data were recorded. In addition, the downloaded expression data of the TNBC transcriptional group included 11 paracancerous tissue samples and 116 TNBC samples that were used for further analysis. In addition, we downloaded the mutation data of 732 ER +/PR + breast cancer subtypes with low immunogenicity. Then, according to TMB index and immune infiltration abundance, ER +/PR + group and TNBC group were compared. As a publicly available database, the relevant information retrieved from TCGA does not require further moral approval.

### Mutant Gene Analysis

The relevant TCGA mutation data were selected using VarScan analysis and triple negative breast cancer mutation gene analysis, and the nonsynonymous mutations and nonsense mutations were included in the measurement of gene mutations. The corresponding TMB value was obtained by calculating the number of tumor mutations per Mb in each sample, and the samples were divided into high and low TMB groups according to the median value of TMB. In addition the survival curve was drawn based on the survival package combined with clinical data to show the correlation between TMB and patient survival.

In addition, because tumors with different gene mutations may have different biological behaviors, we selected representative mutant genes to group TNBC samples (wild and mutant groups) and determined the difference between the two groups using the limma package. Finally, the differences of TMB level and enrichment pathway between wild group and mutant group of 8 genes with high mutation rate were studied and the relationship between these gene mutations and TMB was visualized using the ggpubr package.

### TMB Grouping and Differential Expression Analysis

The TNBC samples containing mutation data and transcriptome data were selected for joint analysis. Using the median of TMB as the critical value, the samples were divided into high and low TMB groups. Then the limma software package was used to determine the difference in the expression of highly mutated genes between high and low TMB groups, and the differential genes were screened with the following cutoff values: P <0.05 and logFC (multiple change) >1.0.

### Analysis of Immune Cell Infiltration in TNBC Samples

Based on 22 different characterization systems of tumor-infiltrating lymphocyte subsets, the deconvolution algorithm CIBERSORT was used to evaluate the relative abundance of immune cells in TNBC samples. The relative content of immune cells in each sample was visualized using the corrplot package, and the correlation between immune cells was analyzed. In addition, the three TNBC samples were divided into wild and mutant groups by target gene, and the difference in immune cell abundance between the two groups was analyzed. The difference between groups was tested using the Wilcoxon test and further explored and visualized using a violin map.

### Analysis of Survival Correlation and Gene Set Enrichment Analysis (GSEA) Functional Enrichment of Target Genes

To further explore the mechanism of the involvement of target genes in the occurrence and progression of the disease and its effect on the prognosis of TNBC, we used the target gene as the cut-in point, grouped its expression median and mutation as cutoff values, and analyzed the pathway enrichment of high and low expression, mutations, and wild samples. We also analyzed the pathway enrichment of high and low TMB samples from TNBC samples. To check the statistical differences between the related pathways, path enrichment analysis was performed using the GSEA software (18). In addition, the survival analysis of the target gene was accomplished by the survival package whereby *P*<0.05 was considered statistically significant.

### Drug Sensitivity Analysis of Target Genes

The drug sensitivity data used in this study were obtained from the CellMiner database (https://discover.nci.nih.gov/cellminer/home.do) (19). First, the transcriptome and drug sensitivity data from the same batch of samples were downloaded, and the drug data and related gene expression data verified by FDA and clinical trials were retained by collating the data. The correlation between target gene expression levels and drug sensitivity was then extracted and further explored using Spearman correlation analysis. The higher the Cor value, the stronger the correlation.

## Results

### Somatic Mutation Landscape in TNBC

**Figure 1:**
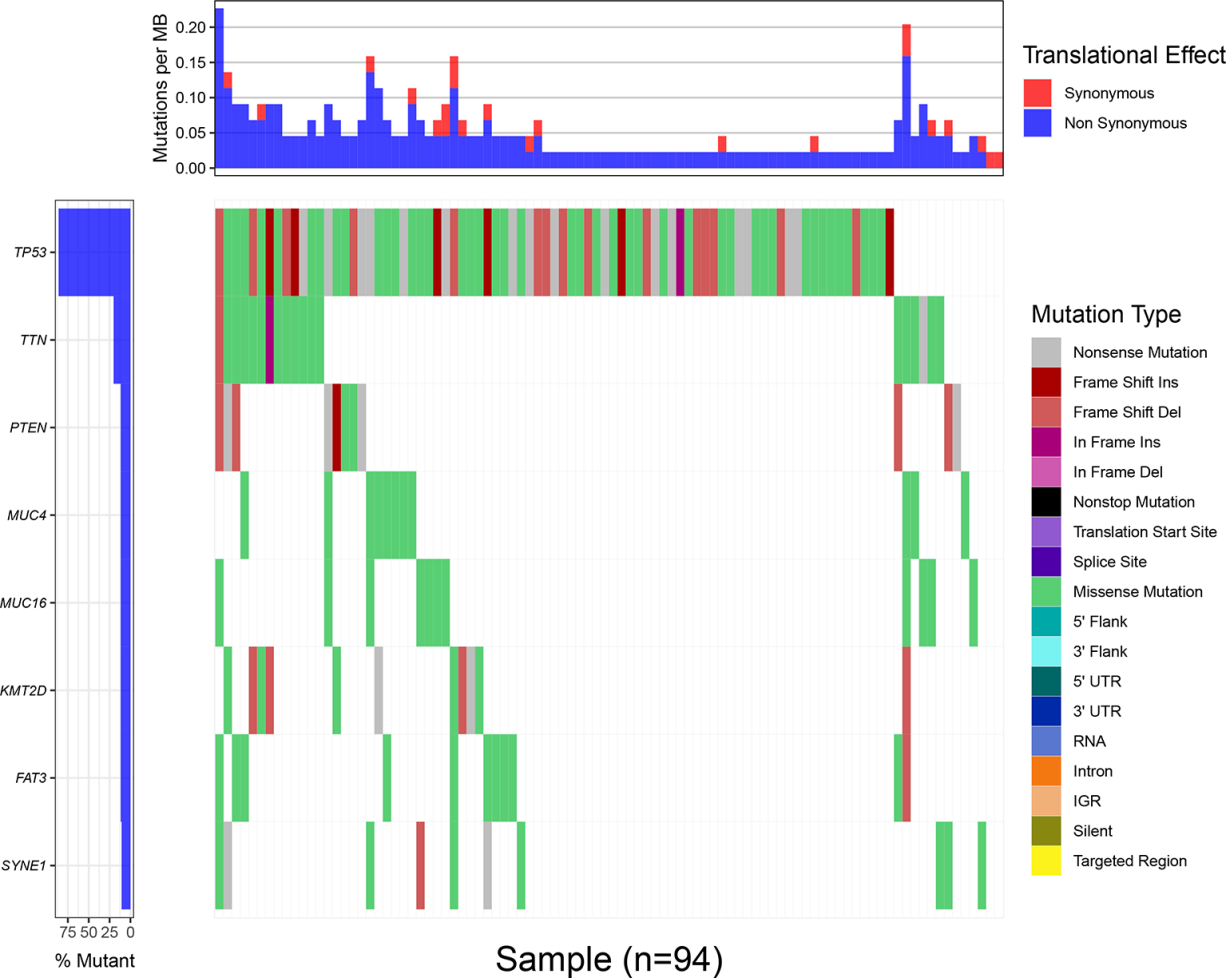
The waterfall map of mutation information of genes in different samples, and different colors represent different types of mutations, showing that only 94 samples out of 101 TNBC mutation samples contained one or more target gene mutations. The upper bar chart shows each MB of each sample Mutation rate, red represents synonymous mutation, blue represents non-synonymous mutation.

Through the statistical analysis of the mutations that cause amino acid changes, 101 cases of TNBC mutations from the TCGA database are shown in **Figure 1**. All the mutations involved are single nucleotide polymorphisms, in which missense mutations are the main type of variation. Under the condition that the number of mutation samples ≥10, eight genes (*TP53, TTN, MUC4, KMT2D, PTEN, FAT3, MUC16*,and *SYNE1*) that showed a high mutation rate in TNBC samples could be screened (**Supplement 1**). The mutation frequency of TP53 (>80%) was the highest among the mutant genes, and the related mutations include missense mutations, such as Frame Shift Ins, Frame Shift Del, In Frame Ins (**Figure 1**).

### Correlation Analysis of TMB

**Figure 2:**
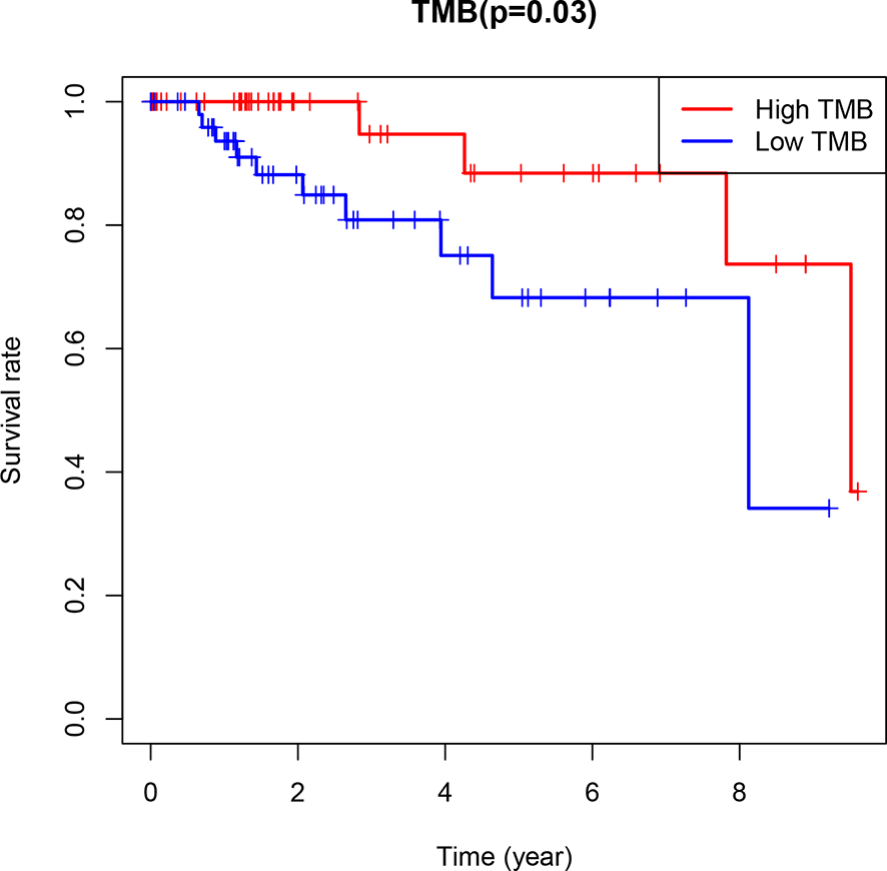
Kaplan-Meier survival analysis with R package was used to analyze the survival of high and low TMB groups (P=0.03<0.05).

The TMB of TNBC samples ranged from 0.05 to 28.03 mutation/Mb (**Supplement 2**), with a median of 1.26 mutation/Mb(The TMB of ER+/PR+ samples is 0.026-118.447 mutation/Mb). Using the median of TMB as the threshold, 101 cases were divided into two categories: the high TMB group (n = 49) and low TMB group (n = 52). Kaplan-Meier survival analysis based on the survival package showed that the 5-year survival rate of the high TMB group was much higher than that of the low TMB group (**Figure 2**). In addition, the grouping analysis of eight genes with high mutation rates revealed a significant difference in TMB levels between the mutant and wild groups, and the TMB levels of the mutant group were higher than those of the wild group in different subgroups (**Supplement 3**).

According to the analysis of the difference between high and low TMB groups, there was a significant difference in the expression of FAT3 among the eight genes with high mutation rates, and FAT3 showed a significantly lower expression trend (logFC=-4.52, *P*=0.0036) in the high TMB group (**Table 1**). Thus, FAT3 served as a target gene in a further analysis of the present study.

**Table 1:** Analysis of differential expression of 8 genes between high and low TMB groups.

Gene	LogFC	pValue
TTN	-0.162458567	0.817215408
PTEN	-0.109935131	0.394175973
SYNE1	-0.111631373	0.790528299
MUC4	-0.60600324	0.154239881
FAT3	-4.519260317	0.003556808
MUC16	0.718850636	0.059164372
TP53	-0.0406174	0.947741078
KMT2D	-0.098513112	0.727527226

### The Relationship Between FAT3 Mutation, TMB, and Immune Infiltration

**Figure 3:**
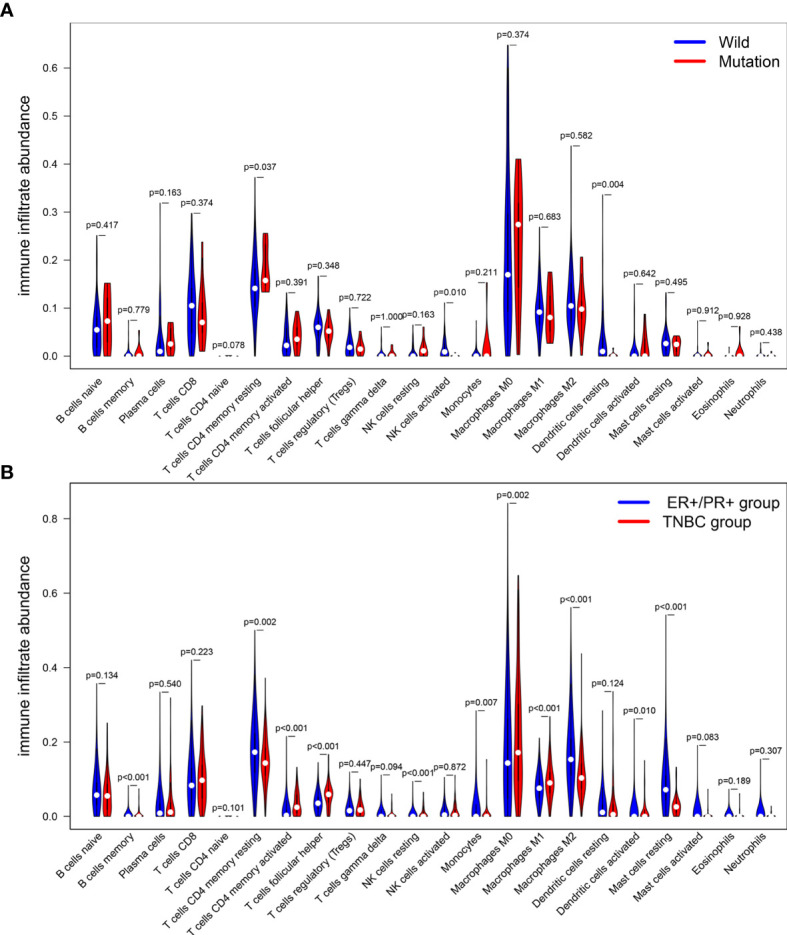
Analysis of the difference of immune cells infiltration abundance. **(A)** between FAT3 mutant group and wild group. **(B)** ER+/PR+ groups and TNBC groups. Blue represents the wild group and red represents the mutant group. The difference was statistically significant (P < 0.05).

**Figure 4:**
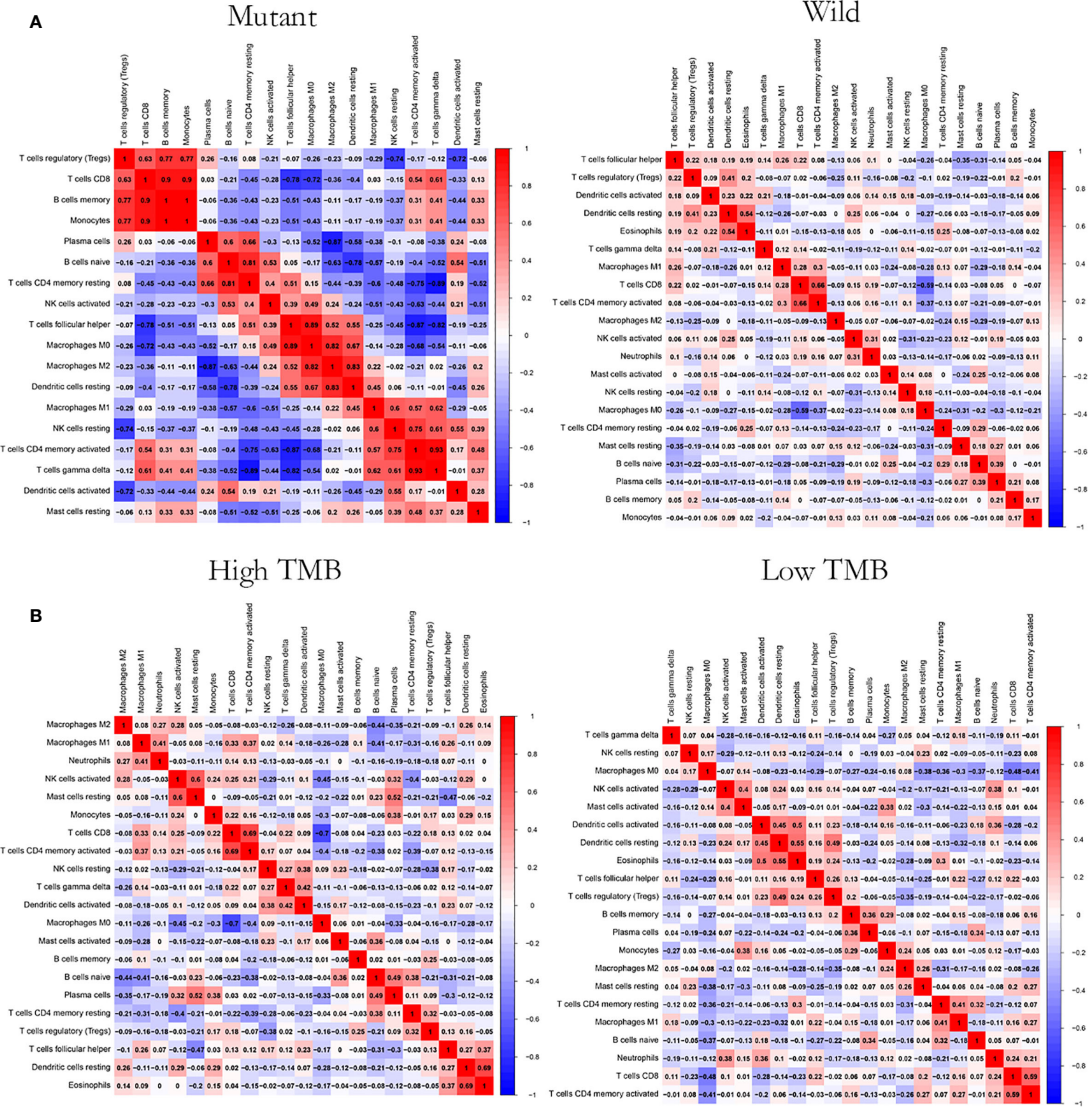
Correlation analysis of immune cell abundance. **(A)** Between the FAT3 mutant groups and the wild groups **(B)** Between high and low TMB groups. Red represents positive correlation and blue represents negative correlation. The greater the absolute value of the correlation coefficient, the greater the correlation.

**Figure 5:**
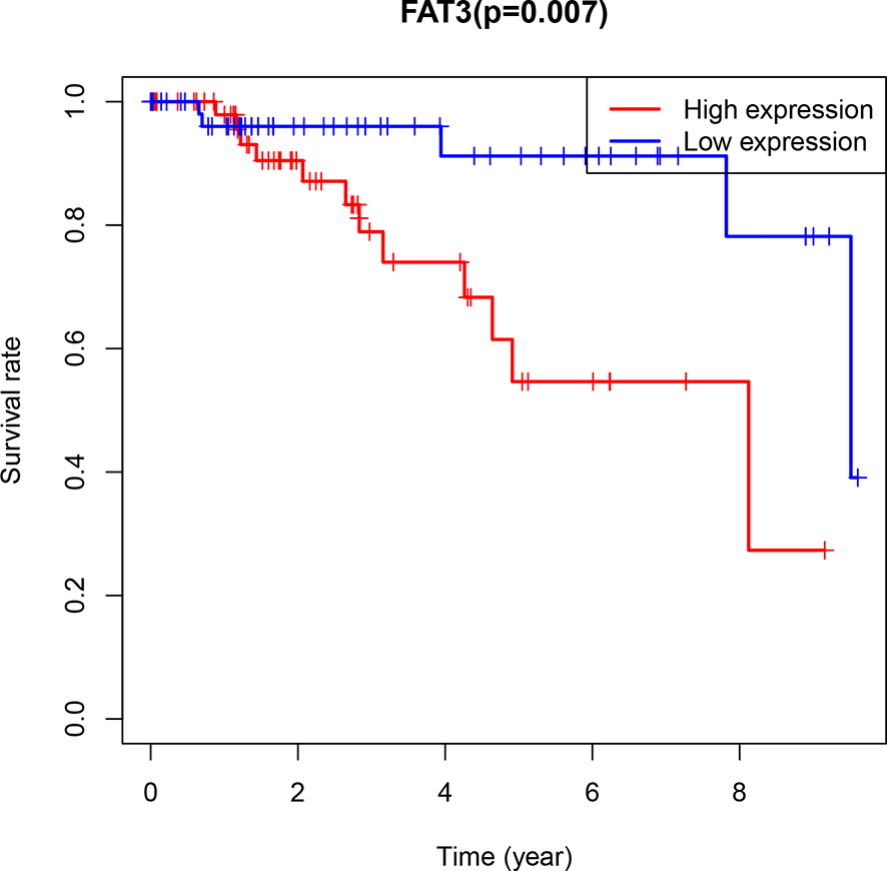
Kaplan-Meier Diagram of FAT3 expression and prognosis in patients. Blue represents low expression, red represents high expression (P=0.007<0.05).

CIBERSOFT algorithm was used to evaluate the immune cell abundance between FAT3 mutation and wild group, TMB high and low group (**Supplement 4**). The results showed that compared with the wild group, the mutant group had more CD4 memory resting T cells, but lesser NK cell activation and dendritic cell resting (**Figure 3A**). And there were significant differences in the abundance of B cells memory, T cells CD4 memory resting, T cells CD4 memory activated, NK cells resting and Mast cells resting in the TNBC group with higher immunogenicity and the ER +/PR + group with lower preimmunogenicity (**Figure 3B**). In addition, the correlation of immune cell abundance revealed a positive or negative feedback relationship between different immune cell contents (**Figure 4**). We also compared the abundance of related immune cells in the high and low TMB groups. Due to limited sample size, the results were not statistically significant, and there was no significant difference in the content of immune cells between the two groups. In addition, survival analysis showed that FAT3 was a mutant gene related to prognosis, and its high expression was significantly correlated with poor prognosis in triple-negative breast cancer patients. Low FAT3 expression may promote better survival (**Figure 5**).

### Pathway Enrichment Analysis

**Figure 6:**
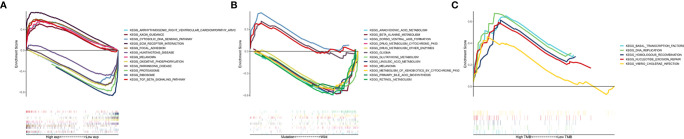
GSEA pathway enrichment analysis among different groups. **(A)** between high and low FAT3 expression groups, **(B)** between FAT3 mutations and wild type groups, **(C)** between high and low TMB groups. Different colors represent different enrichment pathways, Enrichment Score > 0 represents activation of pathways, Enrichment Score < 0 represents inhibition of pathways.

We further studied the functional enrichment of the target gene FAT3. Gene set enrichment analysis using TCGA data and FAT3 as the target gene showed that 34 pathways, including ECM RECEPTOR INTERACTION, TGFBETA SIGNALING PATHWAY, and AXON_GUIDANCE, were significantly enriched in the high expression transcriptome. In the low expression transcriptome group, nine pathways were enriched, including CYTOSOLIC DNA SENSING PATHWAY, RIBOSOME, and OXIDATIVE PHOSPHORYLATION (**Figure 6A**). Interestingly, the results also showed that the three pathways of dorso-ventral axis formation, glioma, and melanoma were significantly enriched in the mutant group with FAT3 as the target gene (**Figure 6B**), while all five pathways were significantly active in the high TMB group, including basal transcription factors, DNA replication, homologous recombination, nucleotide excision repair, and *Vibrio cholerae* infection; no significant active pathway was found in the low TMB group (**Figure 6C**).

### Analysis of the Relationship Between High Mutation Target Gene and Drug Sensitivity in TNBC

According to the analysis of the correlation between target gene and drug sensitivity, a significant correlation was found between the expression levels of the target gene FAT3 and clinical drug sensitivity (**Table 2**), mainly related to drugs such as epothilone B, pelitrexol, asparaginase, methotrexate, and cladribine, and the correlation was of a negative trend; therefore, the lower the expression of FAT3, the more sensitive the cells were to these drugs.

**Table 2:** Analysis of the relationship between the expression level of target gene FAT3 and clinical drug sensitivity.

Gene	Drug	Cor	pValue
FAT3	Epothilone B	-0.585958874	8.72E-07
FAT3	Pelitrexol	-0.572059432	1.80E-06
FAT3	Asparaginase	-0.35910059	0.004836662
FAT3	Methotrexate	-0.333109452	0.009303038
FAT3	Cladribine	-0.322685171	0.011917068
FAT3	Nitrogen mustard	-0.293665411	0.022766073
FAT3	AT-13387	-0.28252021	0.028733379
FAT3	Fludarabine	-0.280942267	0.029675777
FAT3	Cytarabine	-0.274033854	0.034111829
FAT3	Clofarabine	-0.268904736	0.037751184
FAT3	Entinostat	-0.264710586	0.040961052
FAT3	Vorinostat	-0.263182274	0.042185297
FAT3	Parthenolide	-0.256714117	0.047704926

All the drugs presented here are not routinely used in clinic, but are rather effective against breast cancer cell lines in vitro.

## Discussion

Triple-negative breast cancer is a highly heterogeneous and aggressive type of breast cancer. Inhibitors targeting key gene mutations and specific molecular signaling pathways that drive the growth of malignant tumors have been used as single drugs and/or combined with standard chemotherapy regimens (20). Tumor mutation burden and immune cell infiltration are potential biomarkers for cancer treatment and prognosis. Among breast cancer subtypes, TNBC is considered the most immunogenic. Breast cancer immunotherapy based on immune checkpoint inhibitors is currently effective for some TNBCs. These patients with TNBC usually show a high TMB and specific characteristics of immune cell infiltration (21, 22). In this study, to further explore the immunogenicity of TNBC, i) we investigated differences in immune cell infiltration between high and low tumor mutation loads, differences in key pathways, and correlation to the prognosis of TNBC; ii) we analyzed the difference in immune cell infiltration between the wild and mutation group; iii) In order to identify biomarkers related to the prognosis of TNBC, we screened and identified a prognostic gene related to TNBC, the key mutation gene FAT3. With these pursuits, the characteristics of TMB and immune cell infiltration in selected TNBC samples were analyzed.

Recent studies have shown that TMB can be used as the latest independent predictor of the outcome of immune checkpoint inhibitor treatment across multiple tumor types (23). In addition, the tumor mutation burden has the potential to be a prognostic indicator for multiple tumor types (24–26). Using the median TMB as a cutoff line, the TNBC samples were divided into high and low TMB groups. The 5-year survival rate of the high TMB group was much higher than that of the low TMB group. Even without immunotherapy, the survival outcome of the high TMB group is better, consistent with previous research results, our research shows the potential of TMB as a prognostic indicator for patients with TNBC.

Based on 101 triple-negative breast cancer mutation samples, eight genes with higher mutation rates (TP53, TTN, MUC4, KMT2D, PTEN, FAT3, MUC16, and SYNE1) were screened. Among them, TP53 is the most common mutant gene in the cohort, consistent with previous research data (27). In different types of cancer, the unique correlation between TP53 mutation and anti-tumor immunity is the result of the combined effect of TMB and tumor aneuploidy level changes caused by TP53 mutation (28), and in TNBC, patients with TP53 mutations show good immunotherapy response characteristics and thus such mutations may be suitable biomarkers (29). TTN mutations are mostly detected in solid tumors, indicating that these mutations are related to increased TMB and objective response to ICB. In solid tumor studies, the progression-free survival or overall survival of patients with TTN mutations is higher than that of wild-type patients (30). In malignant tumors of the pancreas, ovary, and breast, mutation/overexpression of MUC4 has been fully demonstrated, and the overexpression of MUC4 is involved in trastuzumab resistance (31). KMT2C is one of the most common mutated genes in ER-positive breast cancer (32), whereas KMT2D is mutated in 6% of TNBC. Its regulated genes include RAC3, KRT23, or KRT14 that are involved in cell communication and signal transduction. KMT2D was found to be related to poor survival rate, and low expression of KMT2D at the transcriptional level has prognostic value (33). PTEN is an important phosphatase that plays a role in the interweaving of genetics and epigenetic regulation. PTEN dephosphorylates PtdIns(3,4,5)P 3 to form PtdIns(4,5)P 2 to negatively regulate PI3K/AKT signaling and has been shown to be related to the incidence of breast cancer and tumor progression (34, 35),Consistent with previous research data, TNBC is rich in PTEN mutations and PTEN exhibits low expression at the transcriptome level and is related to treatment resistance and poor survival (36). FAT3, MUC16, and SYNE1 have also been mentioned as common mutant genes in breast cancer in previous studies, but there is no specific report on whether they affect the development and prognosis of breast cancer (37–39).

The research team divided the TNBC samples into a mutant group and a wild group based on eight mutant genes and found that the TMB of the mutant group in each subgroup was higher than that of the wild group.FAT3 encodes atypical cadherins (40); previous studies have shown that FAT3 has detected significant mutations in a variety of malignant tumors, such as breast cancer, lung adenocarcinoma, and small cell lung cancer (37, 41, 42). In the TNBC transcriptome data, FAT3 was the gene with the largest difference between the high and low TMB groups. It was significantly lower expression in the high TMB group, and survival analysis showed that low FAT3 expression predicted better survival. Therefore, when FAT3 is highly mutated, FAT3 expression in TNBC is low, which indicates Patients have better prognosis and survival, which proves that FAT3 can be used as a potential treatment and prognostic biomarker for TNBC. In order to better explore the mechanism of the mutant gene FAT3 in TNBC, we further explored the effect of FAT3 on tumor immune cell abundance and drug sensitivity.

Immune cells in the tumor immune microenvironment play an important role in tumorigenesis, and these tumor-related immune cells can have the function of antagonizing or promoting tumors. Both repair of the tumor microenvironment and restoration of effective immune response are essential to achieving the best therapeutic effect in cancer immunotherapy. Therefore, we need to explore and understand the overall characteristics of the tumor immune microenvironment. Tumor immune cell infiltration is an important component of the tumor immune microenvironment. Compared with the wild group, the FAT3 mutant group had more T cells and CD4 memory resting cells in immune cell infiltration and lower levels of activated NK cells and resting dendritic cells. In research data, resting memory CD4+ T cells are often associated with prognosis of malignant tumor diseases, such as bladder cancer (43), head and neck squamous cell carcinoma (44), and colorectal cancer (45), and it is speculated that FAT3 mutations are related to the infiltration state of resting memory CD4 + T cells, activated NK cells, and resting dendritic cells in triple-negative breast cancer. In addition, in the FAT3 mutation group, a correlation was found between the abundance of various immune cells and TMB level, TMB and immune cell infiltration are closely related to the pathological activities of tumor cells and are related to the prognosis of patients. FAT3 mutations can achieve the value of TNBC biomarkers through the potential impact on both. However, there was no statistical difference in the abundance of immune cells between the high and low TMB groups, which may be due to the small sample size.

Human cells and tissues consist of a complex system network with redundant, convergent, and divergent signaling pathways. Malignant and complex systemic diseases usually involve pathological changes on many levels, including in multiple gene products, signaling pathways, and interconnected signal networks that exist between cells. TNBC is a complex disease with high heterogeneity and effective exploration of signaling pathways can promote in-depth understanding and clinical treatment of the disease. GSEA analysis of selected TNBC samples showed that five pathways were significantly enriched in the high TMB group, including basal transcription factors, DNA replication, homologous recombination, nucleotide excision repair, and Vibrio cholerae infection. Among these, basal transcription factors, DNA replication, homologous recombination, and nucleotide excision repair pathway are involved in cell transcription, recombination, repair, and proliferation. We speculate that this is consistent with previous research data, which may be related to the fact that high TMB predicts good prognosis (46). In the FAT3 mutation samples, three pathways of dorso-ventral axis formation, glioma, and melanoma were significantly enriched.

The multidrug resistance of tumor cells is an important cause for the failure of clinical treatment with anti-tumor drugs. The research team found that FAT3 expression levels are significantly related to clinical sensitivity to certain drugs, including asparaginase and methotrexate. The low expression of FAT3 can enhance the chemotherapy sensitivity of breast cancer cells and can be used as a marker to predict the effect of breast cancer drug treatment.

The research team conducted survival analysis based on the transcriptome data. Among the selected TNBC samples, the survival of the high TMB group was better than that of the low TMB group. Among the mutant genes, FAT3 is the most differentially expressed; low FAT3 expression is associated with good prognosis. FAT3 can be used as a risk predictor for TNBC. Gene enrichment analysis of the high TMB and FAT3 mutant groups showed that both were mostly enriched in pathways related to cell transcription and proliferation. However, the present study has some limitations that must be considered; for example, the legitimate question of whether TMB can be used as an independent risk predictor. In future studies, a large clinical cohort is required to verify the impact of TMB on prognosis. Basic experiments are required to verify the relationship between TMB and immune infiltration.

## Conclusion

Immune cell infiltration and TMB have allowed novel ideas to emerge for the diagnosis, treatment, and prognosis prediction of malignant complex diseases. This study investigated the characteristics of TMB and immune cell infiltration in TNBC and their relationship with prognosis may provide new biomarkers and potential treatment options for TNBC in the future. We found that FAT3 can be used as a risk predictor gene for triple-negative breast cancer and can be used as a target gene for further in-depth research.

## Data Availability Statement

Publicly available datasets were analyzed in this study. This data can be found here: The data that support the findings of this study are available in The Cancer Genome Atlas database at https://cancergenome.nih.gov/.

## Author Contributions

CG, HL and CS conceived and designed the study. XX, JZ, and FF performed data analysis. CL, CZ, LL, and FF contributed analysis tools. CG and HL were the major contributors in writing the manuscript. All authors contributed to the article and approved the submitted version.

## Funding

This work is supported by the grants from National Natural Science Foundation of China (81673799, 81973677) and National Natural Science Foundation of China (81703915).

## Conflict of Interest

The authors declare that the research was conducted in the absence of any commercial or financial relationships that could be construed as a potential conflict of interest.

## Supplementary Material

Supplement 1 Mutations of 8 high mutation rate genes in triple negative breast cancer samples

Supplement 2 The TMB value of each triple negative breast cancer sample and the grouping of the samples.

Supplement 3 Differences in TMB levels of 8 genes between mutant groups and wild groups. Blue represents wild groups, red represents mutant groups, * represents P <0.05, ** represents P <0.01, *** represents P <0.001.

Supplement 4 Evaluation of immune cell abundance in different groups of TNBC samples by CIBERSOFT algorithm. (A) Between the mutant groups and the wild groups (B) Between high and low TMB groups. Different colors represent different immune cell infiltration.

## Abbreviations

TNBC, triple-negative breast cancer; TMB, tumor mutation burden; TCGA, The Cancer Genome Atlas; PD-1, Programmed death-1.
