# Supplementary figures and images for: Tumor Mutation Burden and Immune Invasion Characteristics in Triple Negative Breast Cancer: Genome High-Throughput Data Analysis
Source: Front Immunol. 2021 Apr 21;12:650491. doi: 10.3389/fimmu.2021.650491 (PMC8097167; doi:10.3389/fimmu.2021.650491)

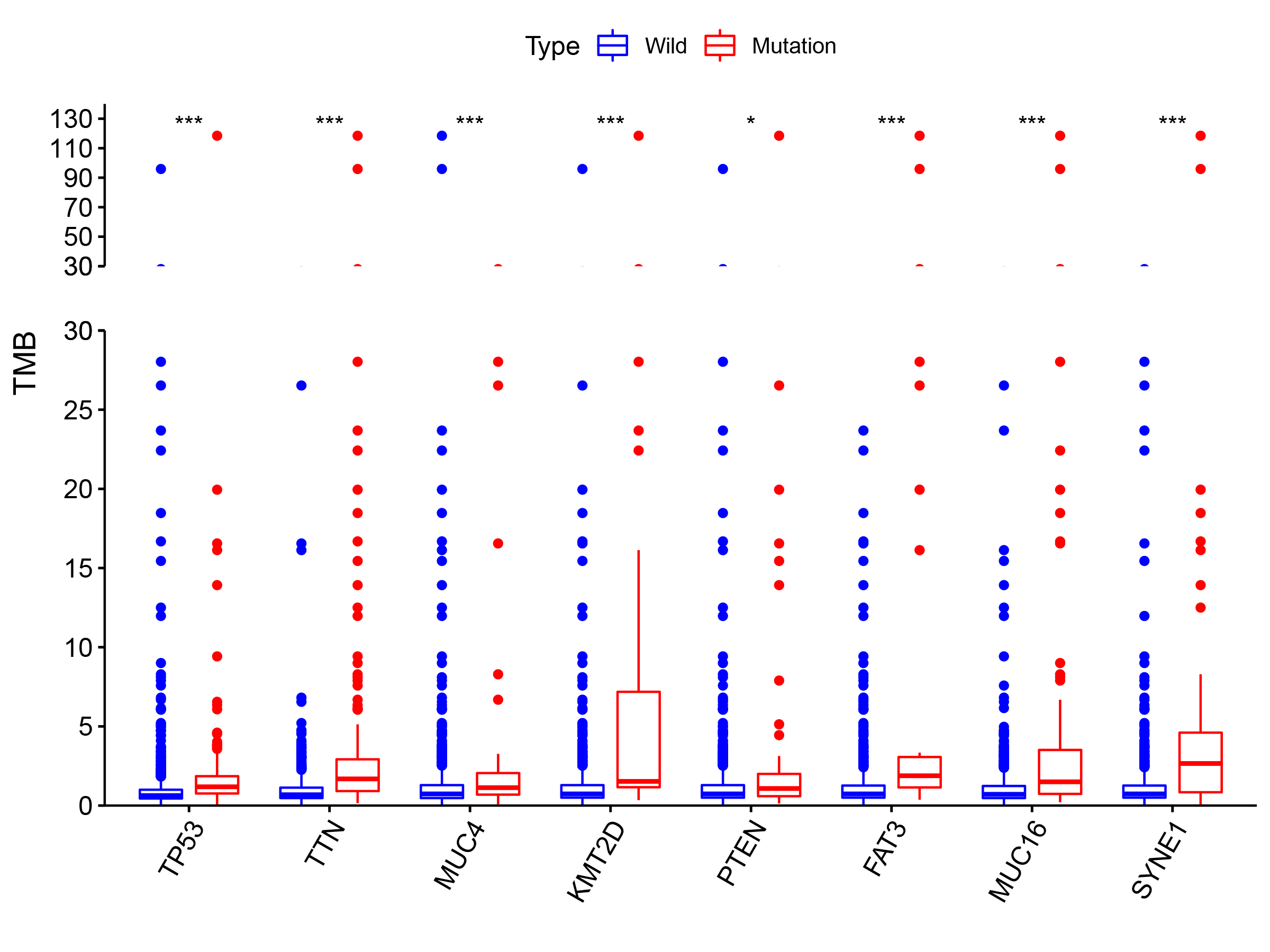

Supplement: Supplement 1 — Mutations of 8 high mutation rate genes in triple negative breast cancer samples [file Image_1.tif]

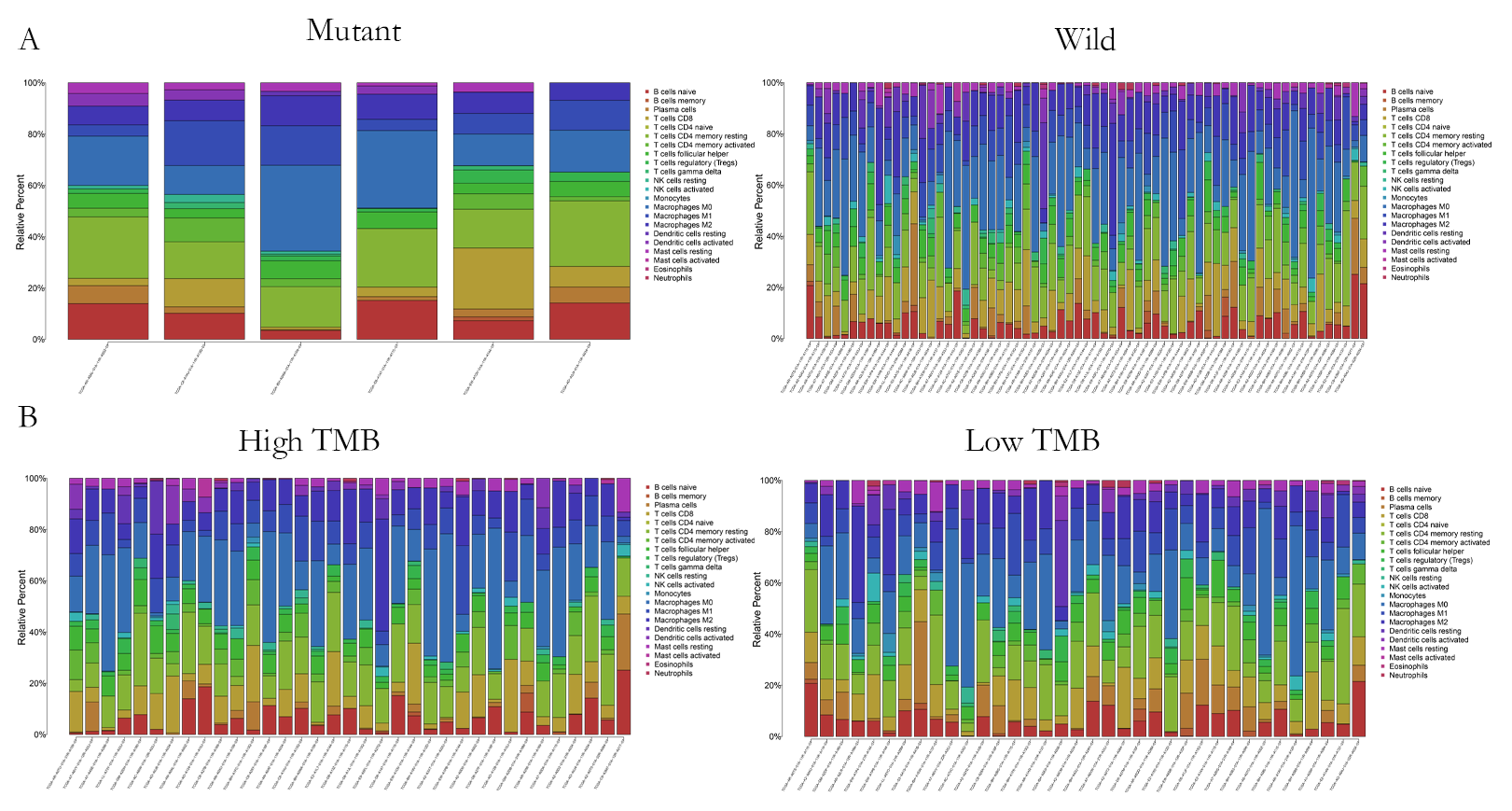

Supplement: Supplement 2 — The TMB value of each triple negative breast cancer sample and the grouping of the samples. [file Image_2.tif]
