# Supplementary material for: Tumor Mutation Burden and Immune Invasion Characteristics in Triple Negative Breast Cancer: Genome High-Throughput Data Analysis
Source: Front Immunol. 2021 Apr 21;12:650491. doi: 10.3389/fimmu.2021.650491 (PMC8097167; doi:10.3389/fimmu.2021.650491)
Supplement: Supplement 3 — Differences in TMB levels of 8 genes between mutant groups and wild groups. Blue represents wild groups, red represents mutant groups, * represents P <0.05, ** represents P <0.01, *** represents P <0.001. [file Table_1.docx]

| Sample | TP53 | TTN | MUC4 | KMT2D | PTEN | FAT3 | MUC16 | SYNE1 |
| --- | --- | --- | --- | --- | --- | --- | --- | --- |
| TCGA-OL-A6VO-01A-12D-A33E-09 | Mutation | Wild | Wild | Wild | Wild | Wild | Wild | Wild |
| TCGA-A2-A04U-01A-11D-A10Y-09 | Mutation | Wild | Wild | Wild | Wild | Wild | Wild | Wild |
| TCGA-GM-A2DB-01A-31D-A19Y-09 | Mutation | Wild | Wild | Wild | Mutation | Wild | Wild | Wild |
| TCGA-E2-A1L7-01A-11D-A142-09 | Mutation | Wild | Wild | Wild | Wild | Wild | Wild | Wild |
| TCGA-BH-A0E0-01A-11W-A071-09 | Mutation | Wild | Wild | Wild | Wild | Wild | Wild | Mutation |
| TCGA-BH-A0RX-01A-21D-A099-09 | Mutation | Wild | Wild | Wild | Wild | Wild | Wild | Wild |
| TCGA-AC-A2BK-01A-11D-A21Q-09 | Mutation | Wild | Wild | Mutation | Wild | Wild | Wild | Wild |
| TCGA-BH-A0BG-01A-11D-A10Y-09 | Mutation | Mutation | Wild | Wild | Wild | Wild | Wild | Wild |
| TCGA-A2-A0D0-01A-11W-A019-09 | Mutation | Wild | Wild | Wild | Wild | Wild | Wild | Wild |
| TCGA-E9-A5FL-01A-11D-A27P-09 | Mutation | Mutation | Wild | Wild | Wild | Wild | Wild | Wild |
| TCGA-A7-A26G-01A-21D-A167-09 | Mutation | Wild | Wild | Wild | Mutation | Wild | Wild | Wild |
| TCGA-BH-A18V-01A-11D-A12B-09 | Mutation | Wild | Wild | Wild | Wild | Wild | Wild | Wild |
| TCGA-EW-A1PH-01A-11D-A14K-09 | Mutation | Wild | Wild | Wild | Wild | Wild | Wild | Wild |
| TCGA-AN-A0AT-01A-11D-A045-09 | Wild | Mutation | Wild | Wild | Wild | Wild | Wild | Mutation |
| TCGA-S3-AA15-01A-11D-A41F-09 | Wild | Wild | Wild | Wild | Wild | Wild | Wild | Wild |
| TCGA-C8-A26X-01A-31D-A16D-09 | Mutation | Mutation | Wild | Mutation | Wild | Wild | Wild | Wild |
| TCGA-E2-A1LL-01A-11D-A142-09 | Wild | Wild | Wild | Wild | Mutation | Wild | Wild | Mutation |
| TCGA-AO-A0J6-01A-11W-A050-09 | Mutation | Mutation | Wild | Mutation | Wild | Wild | Wild | Wild |
| TCGA-EW-A1P8-01A-11D-A142-09 | Mutation | Wild | Wild | Wild | Wild | Wild | Wild | Wild |
| TCGA-BH-A1F6-01A-11D-A13L-09 | Mutation | Wild | Wild | Wild | Wild | Wild | Wild | Wild |
| TCGA-D8-A147-01A-11D-A10Y-09 | Mutation | Mutation | Mutation | Wild | Wild | Mutation | Wild | Wild |
| TCGA-E2-A1LS-01A-12D-A159-09 | Mutation | Wild | Wild | Wild | Wild | Wild | Wild | Wild |
| TCGA-A2-A0T0-01A-22D-A099-09 | Wild | Mutation | Wild | Wild | Wild | Wild | Mutation | Wild |
| TCGA-BH-A42U-01A-12D-A243-09 | Wild | Wild | Wild | Wild | Wild | Wild | Wild | Wild |
| TCGA-E2-A14N-01A-31D-A135-09 | Mutation | Wild | Wild | Wild | Wild | Wild | Wild | Wild |
| TCGA-AC-A2QH-01A-11D-A18P-09 | Mutation | Wild | Mutation | Mutation | Wild | Wild | Wild | Wild |
| TCGA-BH-A0B3-01A-11W-A071-09 | Mutation | Wild | Wild | Wild | Wild | Wild | Wild | Wild |
| TCGA-EW-A1P4-01A-21D-A142-09 | Mutation | Wild | Wild | Wild | Wild | Wild | Wild | Wild |
| TCGA-C8-A1HJ-01A-11D-A13L-09 | Mutation | Wild | Wild | Wild | Wild | Mutation | Wild | Wild |
| TCGA-BH-A1EW-01A-11D-A135-09 | Wild | Wild | Wild | Wild | Wild | Wild | Wild | Wild |
| TCGA-C8-A27B-01A-11D-A167-09 | Mutation | Wild | Wild | Wild | Wild | Wild | Mutation | Mutation |
| TCGA-AR-A2LR-01A-12D-A18P-09 | Mutation | Wild | Wild | Wild | Wild | Wild | Wild | Wild |
| TCGA-A2-A1G6-01A-11D-A13L-09 | Wild | Wild | Wild | Wild | Wild | Wild | Wild | Wild |
| TCGA-AO-A124-01A-11D-A10M-09 | Mutation | Wild | Mutation | Wild | Wild | Wild | Mutation | Mutation |
| TCGA-E2-A1LH-01A-11D-A14G-09 | Mutation | Wild | Wild | Wild | Wild | Wild | Wild | Wild |
| TCGA-S3-AA10-01A-21D-A41F-09 | Mutation | Wild | Wild | Wild | Wild | Wild | Wild | Wild |
| TCGA-D8-A1XQ-01A-11D-A14K-09 | Mutation | Wild | Wild | Mutation | Wild | Mutation | Wild | Mutation |
| TCGA-D8-A13Z-01A-11D-A10Y-09 | Mutation | Wild | Wild | Mutation | Mutation | Wild | Wild | Wild |
| TCGA-EW-A6SB-01A-12D-A32I-09 | Mutation | Wild | Wild | Wild | Wild | Wild | Wild | Wild |
| TCGA-HN-A2NL-01A-11D-A18P-09 | Mutation | Mutation | Wild | Mutation | Wild | Wild | Wild | Wild |
| TCGA-A2-A0SX-01A-12D-A099-09 | Mutation | Wild | Wild | Wild | Wild | Wild | Wild | Wild |
| TCGA-A2-A0T2-01A-11W-A097-09 | Mutation | Wild | Wild | Wild | Wild | Wild | Wild | Wild |
| TCGA-D8-A27M-01A-11D-A16D-09 | Mutation | Wild | Mutation | Wild | Wild | Wild | Wild | Wild |
| TCGA-AR-A1AR-01A-31D-A135-09 | Mutation | Wild | Wild | Wild | Wild | Wild | Wild | Wild |
| TCGA-D8-A1JF-01A-11D-A13L-09 | Mutation | Wild | Mutation | Wild | Wild | Wild | Wild | Wild |
| TCGA-GM-A2DF-01A-11D-A17W-09 | Wild | Wild | Wild | Wild | Wild | Wild | Wild | Wild |
| TCGA-AR-A0TU-01A-31D-A10G-09 | Mutation | Wild | Wild | Wild | Wild | Wild | Wild | Wild |
| TCGA-D8-A143-01A-11D-A10Y-09 | Wild | Wild | Wild | Wild | Wild | Wild | Wild | Wild |
| TCGA-AR-A1AY-01A-21D-A12Q-09 | Mutation | Wild | Wild | Wild | Wild | Wild | Wild | Wild |
| TCGA-A7-A0DA-01A-31D-A10Y-09 | Mutation | Wild | Wild | Wild | Wild | Wild | Wild | Wild |
| TCGA-A2-A3XX-01A-21D-A23C-09 | Mutation | Wild | Wild | Wild | Wild | Wild | Mutation | Wild |
| TCGA-AR-A0U1-01A-11D-A10Y-09 | Mutation | Wild | Wild | Wild | Wild | Wild | Wild | Wild |
| TCGA-E2-A14X-01A-11D-A10Y-09 | Mutation | Mutation | Wild | Wild | Wild | Wild | Wild | Wild |
| TCGA-A7-A4SE-01A-11D-A25Q-09 | Wild | Wild | Wild | Wild | Wild | Wild | Wild | Wild |
| TCGA-AC-A6IW-01A-12D-A33E-09 | Mutation | Wild | Wild | Wild | Wild | Wild | Wild | Wild |
| TCGA-C8-A26Y-01A-11D-A16D-09 | Mutation | Mutation | Wild | Mutation | Mutation | Wild | Wild | Mutation |
| TCGA-C8-A131-01A-11D-A10Y-09 | Mutation | Wild | Wild | Wild | Wild | Wild | Wild | Wild |
| TCGA-BH-A0B9-01A-11W-A071-09 | Mutation | Wild | Wild | Mutation | Wild | Wild | Wild | Wild |
| TCGA-A2-A0D2-01A-21W-A050-09 | Mutation | Wild | Wild | Wild | Wild | Wild | Wild | Wild |
| TCGA-E2-A14R-01A-11D-A10Y-09 | Mutation | Wild | Mutation | Wild | Wild | Wild | Wild | Wild |
| TCGA-AN-A0AR-01A-11W-A019-09 | Mutation | Wild | Wild | Wild | Wild | Wild | Wild | Wild |
| TCGA-AO-A0J4-01A-11W-A050-09 | Mutation | Wild | Wild | Wild | Wild | Mutation | Wild | Wild |
| TCGA-AN-A0XU-01A-11D-A10G-09 | Mutation | Mutation | Wild | Wild | Wild | Wild | Wild | Wild |
| TCGA-AN-A04D-01A-21W-A050-09 | Wild | Mutation | Wild | Wild | Wild | Wild | Mutation | Wild |
| TCGA-GM-A2DH-01A-11D-A17W-09 | Mutation | Wild | Wild | Wild | Wild | Wild | Mutation | Wild |
| TCGA-BH-A0BL-01A-11D-A10Y-09 | Mutation | Wild | Wild | Wild | Wild | Wild | Wild | Wild |
| TCGA-A2-A0CM-01A-31W-A050-09 | Mutation | Wild | Wild | Wild | Wild | Wild | Wild | Wild |
| TCGA-EW-A1OV-01A-11D-A142-09 | Wild | Mutation | Wild | Wild | Mutation | Mutation | Wild | Wild |
| TCGA-BH-A1FC-01A-11D-A13L-09 | Wild | Wild | Wild | Wild | Wild | Wild | Wild | Mutation |
| TCGA-A7-A6VY-01A-12D-A33E-09 | Mutation | Wild | Wild | Wild | Wild | Wild | Wild | Wild |
| TCGA-C8-A3M7-01A-12D-A21Q-09 | Wild | Wild | Mutation | Wild | Wild | Wild | Wild | Wild |
| TCGA-D8-A1XK-01A-21D-A14K-09 | Mutation | Mutation | Wild | Wild | Mutation | Mutation | Mutation | Mutation |
| TCGA-A1-A0SK-01A-12D-A099-09 | Mutation | Wild | Mutation | Wild | Wild | Mutation | Wild | Wild |
| TCGA-A1-A0SP-01A-11D-A099-09 | Mutation | Wild | Wild | Wild | Wild | Wild | Wild | Wild |
| TCGA-A8-A09X-01A-11W-A019-09 | Mutation | Wild | Wild | Wild | Wild | Wild | Wild | Wild |
| TCGA-A2-A0YE-01A-11D-A10G-09 | Mutation | Wild | Wild | Wild | Wild | Wild | Wild | Wild |
| TCGA-A7-A6VW-01A-21D-A33E-09 | Mutation | Wild | Wild | Wild | Wild | Wild | Wild | Wild |
| TCGA-LL-A5YO-01A-21D-A28B-09 | Mutation | Wild | Wild | Wild | Wild | Wild | Wild | Wild |
| TCGA-A2-A3XT-01A-11D-A22X-09 | Mutation | Mutation | Wild | Wild | Wild | Wild | Wild | Wild |
| TCGA-E2-A150-01A-11D-A12B-09 | Mutation | Wild | Wild | Wild | Wild | Wild | Wild | Wild |
| TCGA-AO-A12F-01A-11D-A10Y-09 | Wild | Wild | Wild | Wild | Wild | Wild | Wild | Wild |
| TCGA-AR-A0TS-01A-11D-A10Y-09 | Mutation | Wild | Wild | Wild | Wild | Wild | Wild | Wild |
| TCGA-A8-A08R-01A-11W-A050-09 | Mutation | Wild | Wild | Wild | Wild | Mutation | Wild | Mutation |
| TCGA-AR-A256-01A-11D-A167-09 | Mutation | Wild | Wild | Wild | Wild | Wild | Mutation | Wild |
| TCGA-AQ-A04J-01A-02W-A050-09 | Mutation | Wild | Wild | Wild | Wild | Wild | Wild | Wild |
| TCGA-BH-A0WA-01A-11D-A10G-09 | Mutation | Wild | Wild | Wild | Wild | Mutation | Wild | Wild |
| TCGA-C8-A12V-01A-11D-A10Y-09 | Wild | Wild | Wild | Wild | Mutation | Wild | Wild | Wild |
| TCGA-E2-A158-01A-11D-A12B-09 | Mutation | Mutation | Wild | Wild | Wild | Wild | Wild | Wild |
| TCGA-AO-A1KR-01A-12D-A142-09 | Wild | Wild | Wild | Wild | Wild | Wild | Wild | Wild |
| TCGA-AN-A0G0-01A-11W-A050-09 | Mutation | Wild | Wild | Wild | Wild | Wild | Wild | Wild |
| TCGA-D8-A1JL-01A-11D-A13L-09 | Mutation | Wild | Wild | Wild | Wild | Wild | Wild | Wild |
| TCGA-A7-A6VV-01A-22D-A33E-09 | Mutation | Wild | Wild | Wild | Wild | Wild | Wild | Wild |
| TCGA-EW-A1PB-01A-11D-A142-09 | Mutation | Wild | Wild | Wild | Wild | Wild | Wild | Wild |
| TCGA-AN-A0AL-01A-11W-A019-09 | Mutation | Mutation | Wild | Wild | Mutation | Mutation | Wild | Wild |
| TCGA-LL-A73Y-01A-11D-A33E-09 | Wild | Mutation | Mutation | Wild | Wild | Wild | Wild | Wild |
| TCGA-AO-A128-01A-11D-A10M-09 | Mutation | Wild | Mutation | Wild | Mutation | Wild | Mutation | Wild |
| TCGA-AR-A5QQ-01A-11D-A28B-09 | Mutation | Wild | Wild | Wild | Mutation | Wild | Wild | Wild |
| TCGA-D8-A27F-01A-11D-A16D-09 | Mutation | Wild | Wild | Wild | Wild | Wild | Wild | Wild |
| TCGA-AC-A7VC-01A-11D-A351-09 | Mutation | Wild | Wild | Mutation | Wild | Wild | Wild | Wild |
| TCGA-A8-A07O-01A-11W-A019-09 | Wild | Wild | Wild | Wild | Wild | Wild | Mutation | Wild |
| TCGA-BH-A18G-01A-11D-A12B-09 | Wild | Mutation | Mutation | Mutation | Wild | Mutation | Mutation | Wild |

Supplement 1：Mutations of 8 high mutation rate genes in triple negative breast cancer samples
