# Supplementary material for: Tumor Mutation Burden and Immune Invasion Characteristics in Triple Negative Breast Cancer: Genome High-Throughput Data Analysis
Source: Front Immunol. 2021 Apr 21;12:650491. doi: 10.3389/fimmu.2021.650491 (PMC8097167; doi:10.3389/fimmu.2021.650491)
Supplement: Supplement 4 — Evaluation of immune cell abundance in different groups of TNBC samples by CIBERSOFT algorithm. (A) Between the mutant groups and the wild groups (B) Between high and low TMB groups. Different colors represent different immune cell infiltration. [file Table_2.docx]

| id | TMB | Group |
| --- | --- | --- |
| TCGA-BH-A18G-01A-11D-A12B-09 | 28.02632 | High TMB |
| TCGA-D8-A1XK-01A-21D-A14K-09 | 19.94737 | High TMB |
| TCGA-AO-A128-01A-11D-A10M-09 | 16.55263 | High TMB |
| TCGA-D8-A1XQ-01A-11D-A14K-09 | 16.13158 | High TMB |
| TCGA-C8-A26Y-01A-11D-A16D-09 | 13.92105 | High TMB |
| TCGA-AR-A256-01A-11D-A167-09 | 3.868421 | High TMB |
| TCGA-BH-A18V-01A-11D-A12B-09 | 3.763158 | High TMB |
| TCGA-A2-A0T0-01A-22D-A099-09 | 3.394737 | High TMB |
| TCGA-A8-A08R-01A-11W-A050-09 | 3.342105 | High TMB |
| TCGA-E2-A1LH-01A-11D-A14G-09 | 3.105263 | High TMB |
| TCGA-A7-A0DA-01A-31D-A10Y-09 | 3.105263 | High TMB |
| TCGA-AO-A124-01A-11D-A10M-09 | 3.052632 | High TMB |
| TCGA-C8-A27B-01A-11D-A167-09 | 2.973684 | High TMB |
| TCGA-D8-A147-01A-11D-A10Y-09 | 2.868421 | High TMB |
| TCGA-GM-A2DH-01A-11D-A17W-09 | 2.789474 | High TMB |
| TCGA-EW-A1PB-01A-11D-A142-09 | 2.657895 | High TMB |
| TCGA-AN-A0AT-01A-11D-A045-09 | 2.552632 | High TMB |
| TCGA-C8-A3M7-01A-12D-A21Q-09 | 2.526316 | High TMB |
| TCGA-E2-A14R-01A-11D-A10Y-09 | 2.447368 | High TMB |
| TCGA-GM-A2DB-01A-31D-A19Y-09 | 2.421053 | High TMB |
| TCGA-AN-A0AR-01A-11W-A019-09 | 2.394737 | High TMB |
| TCGA-AO-A0J6-01A-11W-A050-09 | 2.368421 | High TMB |
| TCGA-S3-AA10-01A-21D-A41F-09 | 2.368421 | High TMB |
| TCGA-LL-A73Y-01A-11D-A33E-09 | 2.315789 | High TMB |
| TCGA-AC-A6IW-01A-12D-A33E-09 | 2.289474 | High TMB |
| TCGA-BH-A1FC-01A-11D-A13L-09 | 2.263158 | High TMB |
| TCGA-C8-A1HJ-01A-11D-A13L-09 | 2.263158 | High TMB |
| TCGA-AN-A0XU-01A-11D-A10G-09 | 2.210526 | High TMB |
| TCGA-A7-A6VY-01A-12D-A33E-09 | 2.105263 | High TMB |
| TCGA-D8-A1JL-01A-11D-A13L-09 | 2.078947 | High TMB |
| TCGA-EW-A1OV-01A-11D-A142-09 | 1.947368 | High TMB |
| TCGA-AN-A04D-01A-21W-A050-09 | 1.894737 | High TMB |
| TCGA-E2-A158-01A-11D-A12B-09 | 1.894737 | High TMB |
| TCGA-A7-A4SE-01A-11D-A25Q-09 | 1.868421 | High TMB |
| TCGA-AO-A0J4-01A-11W-A050-09 | 1.815789 | High TMB |
| TCGA-E9-A5FL-01A-11D-A27P-09 | 1.815789 | High TMB |
| TCGA-BH-A0WA-01A-11D-A10G-09 | 1.789474 | High TMB |
| TCGA-A2-A0D2-01A-21W-A050-09 | 1.763158 | High TMB |
| TCGA-AN-A0AL-01A-11W-A019-09 | 1.763158 | High TMB |
| TCGA-HN-A2NL-01A-11D-A18P-09 | 1.657895 | High TMB |
| TCGA-D8-A13Z-01A-11D-A10Y-09 | 1.657895 | High TMB |
| TCGA-E2-A1LS-01A-12D-A159-09 | 1.578947 | High TMB |
| TCGA-A2-A3XT-01A-11D-A22X-09 | 1.552632 | High TMB |
| TCGA-AC-A2BK-01A-11D-A21Q-09 | 1.5 | High TMB |
| TCGA-AR-A0TU-01A-31D-A10G-09 | 1.5 | High TMB |
| TCGA-AR-A0U1-01A-11D-A10Y-09 | 1.447368 | High TMB |
| TCGA-D8-A27F-01A-11D-A16D-09 | 1.315789 | High TMB |
| TCGA-A2-A0D0-01A-11W-A019-09 | 1.315789 | High TMB |
| TCGA-E2-A1L7-01A-11D-A142-09 | 1.315789 | High TMB |
| TCGA-A7-A6VW-01A-21D-A33E-09 | 1.263158 | Low TMB |
| TCGA-A1-A0SK-01A-12D-A099-09 | 1.263158 | Low TMB |
| TCGA-EW-A1P4-01A-21D-A142-09 | 1.236842 | Low TMB |
| TCGA-AR-A1AY-01A-21D-A12Q-09 | 1.236842 | Low TMB |
| TCGA-E2-A14N-01A-31D-A135-09 | 1.236842 | Low TMB |
| TCGA-BH-A0BL-01A-11D-A10Y-09 | 1.210526 | Low TMB |
| TCGA-C8-A26X-01A-31D-A16D-09 | 1.210526 | Low TMB |
| TCGA-AR-A2LR-01A-12D-A18P-09 | 1.157895 | Low TMB |
| TCGA-AR-A1AR-01A-31D-A135-09 | 1.157895 | Low TMB |
| TCGA-C8-A131-01A-11D-A10Y-09 | 1.157895 | Low TMB |
| TCGA-D8-A27M-01A-11D-A16D-09 | 1.131579 | Low TMB |
| TCGA-AR-A5QQ-01A-11D-A28B-09 | 1.105263 | Low TMB |
| TCGA-A2-A04U-01A-11D-A10Y-09 | 1.105263 | Low TMB |
| TCGA-AO-A1KR-01A-12D-A142-09 | 1.078947 | Low TMB |
| TCGA-AQ-A04J-01A-02W-A050-09 | 1.052632 | Low TMB |
| TCGA-D8-A1JF-01A-11D-A13L-09 | 1.052632 | Low TMB |
| TCGA-EW-A6SB-01A-12D-A32I-09 | 1.026316 | Low TMB |
| TCGA-A8-A09X-01A-11W-A019-09 | 1.026316 | Low TMB |
| TCGA-D8-A143-01A-11D-A10Y-09 | 1.026316 | Low TMB |
| TCGA-E2-A150-01A-11D-A12B-09 | 0.973684 | Low TMB |
| TCGA-EW-A1PH-01A-11D-A14K-09 | 0.947368 | Low TMB |
| TCGA-A7-A6VV-01A-22D-A33E-09 | 0.921053 | Low TMB |
| TCGA-A2-A0CM-01A-31W-A050-09 | 0.894737 | Low TMB |
| TCGA-AR-A0TS-01A-11D-A10Y-09 | 0.868421 | Low TMB |
| TCGA-A8-A07O-01A-11W-A019-09 | 0.842105 | Low TMB |
| TCGA-A7-A26G-01A-21D-A167-09 | 0.842105 | Low TMB |
| TCGA-BH-A0B9-01A-11W-A071-09 | 0.789474 | Low TMB |
| TCGA-AN-A0G0-01A-11W-A050-09 | 0.763158 | Low TMB |
| TCGA-BH-A0BG-01A-11D-A10Y-09 | 0.763158 | Low TMB |
| TCGA-A1-A0SP-01A-11D-A099-09 | 0.763158 | Low TMB |
| TCGA-E2-A1LL-01A-11D-A142-09 | 0.736842 | Low TMB |
| TCGA-BH-A0E0-01A-11W-A071-09 | 0.736842 | Low TMB |
| TCGA-AO-A12F-01A-11D-A10Y-09 | 0.710526 | Low TMB |
| TCGA-EW-A1P8-01A-11D-A142-09 | 0.710526 | Low TMB |
| TCGA-A2-A0YE-01A-11D-A10G-09 | 0.657895 | Low TMB |
| TCGA-BH-A0B3-01A-11W-A071-09 | 0.631579 | Low TMB |
| TCGA-A2-A3XX-01A-21D-A23C-09 | 0.605263 | Low TMB |
| TCGA-GM-A2DF-01A-11D-A17W-09 | 0.605263 | Low TMB |
| TCGA-BH-A1F6-01A-11D-A13L-09 | 0.605263 | Low TMB |
| TCGA-AC-A2QH-01A-11D-A18P-09 | 0.578947 | Low TMB |
| TCGA-A2-A0T2-01A-11W-A097-09 | 0.526316 | Low TMB |
| TCGA-LL-A5YO-01A-21D-A28B-09 | 0.526316 | Low TMB |
| TCGA-A2-A0SX-01A-12D-A099-09 | 0.473684 | Low TMB |
| TCGA-E2-A14X-01A-11D-A10Y-09 | 0.447368 | Low TMB |
| TCGA-BH-A0RX-01A-21D-A099-09 | 0.447368 | Low TMB |
| TCGA-C8-A12V-01A-11D-A10Y-09 | 0.394737 | Low TMB |
| TCGA-BH-A1EW-01A-11D-A135-09 | 0.394737 | Low TMB |
| TCGA-AC-A7VC-01A-11D-A351-09 | 0.342105 | Low TMB |
| TCGA-OL-A6VO-01A-12D-A33E-09 | 0.289474 | Low TMB |
| TCGA-S3-AA15-01A-11D-A41F-09 | 0.184211 | Low TMB |
| TCGA-BH-A42U-01A-12D-A243-09 | 0.131579 | Low TMB |
| TCGA-A2-A1G6-01A-11D-A13L-09 | 0.052632 | Low TMB |

Supplement 2：The TMB value of each triple negative breast cancer sample and the grouping of the samples.
